# Direct effect of dasatinib on signal transduction pathways associated with a rapid mobilization of cytotoxic lymphocytes

**DOI:** 10.1002/cam4.925

**Published:** 2016-10-10

**Authors:** Noriyoshi Iriyama, Yoshihiro Hatta, Masami Takei

**Affiliations:** ^1^ Division of Hematology and RheumatologyDepartment of MedicineNihon University School of MedicineTokyoJapan

**Keywords:** AKT, cytotoxic lymphocyte, dasatinib, ERK, mobilization

## Abstract

It has been shown that an increase in cytotoxic lymphocyte counts in the peripheral blood occurs rapidly after taking dasatinib, but the underlying mechanism is not yet elucidated. To investigate the influence of dasatinib on signal transduction pathways, we investigated the changes in JAK‐STAT, mitogen‐activated protein kinase (MAPK), and AKT in cytotoxic lymphocytes, including natural killer (NK) cells and cytotoxic T lymphocytes (CTLs), before and after dasatinib treatment in chronic myeloid leukemia patients. Among a total of 30 patients, 18 were treated with dasatinib, nine with imatinib, and three with nilotinib. At constitutive levels, the expression of phosphorylated proteins, pSTAT1, pSTAT3, and pERK in NK cells and pSTAT3 in CTLs, was significantly higher in dasatinib‐treated patients. Among the patients evaluated, only dasatinib‐treated patients showed inhibition of multiple signaling pathways after taking a tyrosine kinase inhibitor. The magnitude of pERK and pAKT inhibition was closely associated with an increase in NK cells and CTLs, respectively, after taking a tyrosine kinase inhibitor. Those responses were more evident in patients with cytomegalovirus IgG positivity. In this study, we demonstrated for the first time, the influence of dasatinib on cell events in cytotoxic lymphocytes in vivo and explained the possible underlying mechanism that results in lymphocyte mobilization after dasatinib treatment.

## Introduction

The approval of the tyrosine kinase inhibitor (TKI), imatinib, markedly improved the prognoses of patients with chronic myeloid leukemia (CML) and Philadelphia chromosome‐positive acute lymphoblastic leukemia (Ph+ ALL) 1, 2, 3, 4. Dasatinib, a multikinase inhibitor more potently active for ABL inhibition than imatinib, has also been approved as a treatment agent for CML and Ph+ ALL 5, 6. In addition to an ABL inhibitory effect, dasatinib exerts unique effects on the immune system such as increased incidence of lymphocytosis and regulatory T‐cell (Treg) inhibition 7, 8, 9, 10, 11. The phenomenon is specific to dasatinib, and patients with lymphocytosis are predicted to achieve more favorable treatment results in Ph+ leukemias 7, 8, 9. The proliferated lymphocytes have large granular lymphocyte morphology and show a differentiated natural killer (NK) cell or cytotoxic T‐lymphocyte (CTL) immunophenotype 7, 8, 9, 10. A recent study indicated that NK cells derived from dasatinib‐treated patients who had lymphocytosis showed higher toxicity against the CML cell line K562 12. Interestingly, the incidence of lymphocytosis is possibly affected by a prior history of cytomegalovirus (CMV) infection as indicated by CMV IgG status, with a higher incidence rate in patients who are positive for CMV IgG 13. In contrast, CMV‐related complications such as colitis or CMV reactivation are also reported in Ph+ leukemias 7, 13. Thus, the actual influence on the immune system by dasatinib has been consolidated. However, specific evidence that explains how dasatinib interacts with the immune system in the body is still not available.

Importantly, recent reports have documented that lymphocytosis occurs within 1‐2 h after dasatinib intake, indicating a rapid mobilization of pooled lymphocytes 12, 14. Although substantial evidence demonstrating the contribution of immune activation involving NK cells and cytotoxic T lymphocytes (CTLs) on dasatinib treatment has accumulated, there has been no research explaining the mechanism underlying the rapid response to dasatinib in vivo. It is well known that the activity, adhesion, and migration of lymphocytes are regulated through changes in multiple signal transduction pathways such as the mitogen‐activated protein kinase (MAPK) and AKT pathways 15, 16. When the speed of response is considered, the changes in signal transduction pathways not involving protein synthesis or protein degradation are assumed to be critical regulation systems for rapid mobilization of cytotoxic lymphocytes. However, samples procured from patients are complex, and therefore, the evaluation of changes in cell signaling limited to specific subpopulations such as NK cells or CTLs is difficult when conventional methods such as western blots are used.

Phospho‐flow, a unique method that enables the sensitive and quantitative detection of phosphorylated proteins at the single‐cell level, was developed by the Nolan group recently 17, 18. This method is able to evaluate cell signaling in each lymphocyte type, because total nuclear cells can be separated by costaining with antibodies for their surface markers. Given the lack of detailed data regarding the effect of dasatinib on the normal immune system, we applied the phospho‐flow method to perform a comprehensive evaluation of the changes in signal transduction pathways in lymphocytes of CML patients during TKI treatment. Furthermore, the association between the change in the expression of phosphorylated proteins and the NK cell or CTL mobilization was investigated. Herein, we describe how dasatinib may influence cell signaling in cytotoxic lymphocytes and describe the possible mechanism that results in the activation and mobilization of cytotoxic lymphocytes.

## Patients and Methods

### Patients and treatment

This study included well‐controlled CML patients in the chronic phase, who scored less than 1% of the International Scale (IS) (to avoid the effect of concomitant mononuclear cells derived from the CML clone) and who were treated for more than 3 months with any TKI, because protein phosphorylation and response to cytokines in immune cells are impaired at the diagnostic stage 19. Patients who were taking any cytotoxic agent or immunosuppressant (e.g., corticosteroid), had chronic inflammatory disease, or who had received allogeneic hematopoietic transplantation were excluded from the analysis. The background of the patients in the study was primarily immunocompetent with little or no effect of the underlying disease. The study was approved by the research ethics board of Nihon University Itabashi Hospital (identifier: RK‐150609‐15), and the study was conducted in accordance with the Declaration of Helsinki. All patients gave written informed consent prior to study enrollment.

### Assessment of treatment response and molecular analyses

Quantification of the *BCR‐ABL1* transcript by real‐time quantitative polymerase chain reaction analysis was performed to assess the molecular response. *BCR‐ABL1* transcript analysis was performed by Special Reference Laboratories (SRL; Tokyo, Japan). A major molecular response (MMR) was defined as ≤0.1% and a deep molecular response (DMR) was ≤0.0032% of the IS.

### Reagents

Dasatinib was purchased from BioVision (Mountain View, CA) and dissolved in dimethyl sulfoxide (DMSO) at a concentration of 300 *μ*mol/L. Imatinib and nilotinib were purchased from Tokyo Chemical Industry (Tokyo, Japan) and ChemScene (Monmouth Junction, NJ) and dissolved in DMSO at a concentration of 2 mmol/L, respectively.

Antibodies for immunophenotyping, including phycoerythrin (PE)‐conjugated anti‐CD56 IgG, PC5‐conjugated anti‐CD8 IgG, PC7‐conjugated anti‐CD3 IgG, and each isotype control IgG were purchased from Beckman Coulter (Brea, CA). Fluorescein isothiocyanate (FITC)‐labeled polyclonal rabbit IgG antibodies for phosphorylated proteins, including pJAK1 (Y1034, #3238R), pJAK2 (Y1007, #2485R), pSTAT1 (Y701, #1657R), pSTAT3 (Y705, #1658R), pERK (T202/Y204, #1646R), pJNK (T183/Y185, #1640R), pp38 (T180/Y182, #2210R), pAKT (Y315, #5193R), and isotype control (#0295P) were purchased from Bioss (Wobun, MA, USA).

### Sample collection

Peripheral blood samples were collected before and after TKI intake. Sample collection after TKI dosage was performed 1 h after dasatinib treatment or 2 h after imatinib or nilotinib treatment, which is considered the peak concentration time as described previously 12. Complete blood cell counts and the percentage of each leukocyte subset were evaluated in all samples. For analysis of signal transduction pathways using the phospho‐flow method, mononuclear cell fractions were isolated from freshly collected peripheral blood using Lymphoprep^™^ (Cosmo Bio Co., Ltd., Tokyo, Japan) to classify each cellular subset precisely by reducing the effect of outliers.

### Cell culture

Mononuclear cells obtained from patient blood samples were cultured in a RPMI‐1640 medium (Gibco‐BRL, Rockville, MD) supplemented with 10% heat‐inactivated fetal bovine serum (FBS) (Gibco‐BRL) and antibiotics [100 U/mL penicillin and 100 *μ*g/mL streptomycin (Gibco‐BRL)] at 37°C in a humidified atmosphere (5% CO_2_ in air) with 100 nmol/L and 300 nmol/L dasatinib, 2 *μ*mol/L imatinib, and 2 *μ*mol/L nilotinib, for 60 min. DMSO was used as a control vehicle.

### Phospho‐flow method

Mononuclear cells isolated from patient peripheral blood samples were fixed with 1.5% paraformaldehyde (PFA) at room temperature for 10 min and were permeabilized with 90% ice‐cold methanol at −20°C overnight. For analysis of phosphorylated proteins in combination with surface antigen staining, we first washed the cells twice with phosphate‐buffered saline (PBS) containing 2.5% FBS and 0.5% NaN_3_ (PBSF) and then stained them with surface antibodies including CD3, CD8, and CD56 at room temperature for 15 min in the dark. Cells stained with antibodies were divided and stained with each phospho‐specific antibody at a concentration of 10 *μ*g/mL at room temperature for 30 min in the dark. After staining, cells were rewashed with PBSF and analyzed by flow cytometer (FC500, Beckman Coulter). The absolute lymphocyte count was expressed using the total blood cell count and the percentage of lymphocytes. Lymphocytes were classified according to the immunophenotypes determined by flow cytometry. The lymphocyte fraction was gated with forward and side scatter, and double‐gating analyses were performed with CD3*CD8 and CD3*CD56. The lineages of CTLs were defined according to expression of CD3^+^ CD8^+^ and NK cells with CD3‐CD56^+^. The absolute values of individual lymphocyte subsets were expressed as the total lymphocyte count multiplied by the percentage of each lymphocyte subset. We found that the fixation/permeabilization step had little or no effect on the probability of each lymphocyte fraction. For evaluation of phospho‐specific proteins, NK cell and CTL fractions were gated separately and the expression level of each protein in the cells specified was denoted as “median” fluorescence intensity (MFI), as is recommended for the assessment of blood samples 18. The changes in MFI values are presented as log2 (fold change [FC]). The Flow‐Check^™^ system (Beckman Coulter) was used for ensuring the quality of values obtained in daily analyses.

### Statistical analysis

The Mann–Whitney U and ANOVA tests were used to determine statistical significance in each group as appropriate. The correlation between factors was evaluated with the Pearson correlation coefficient. *P *<* *0.05 was considered to be significant. Statistical analyses were performed using JMP software, version 11.0.0 (SAS Institute Inc., Cary, NC).

## Results

### Patient characteristics

**Figure 1:**
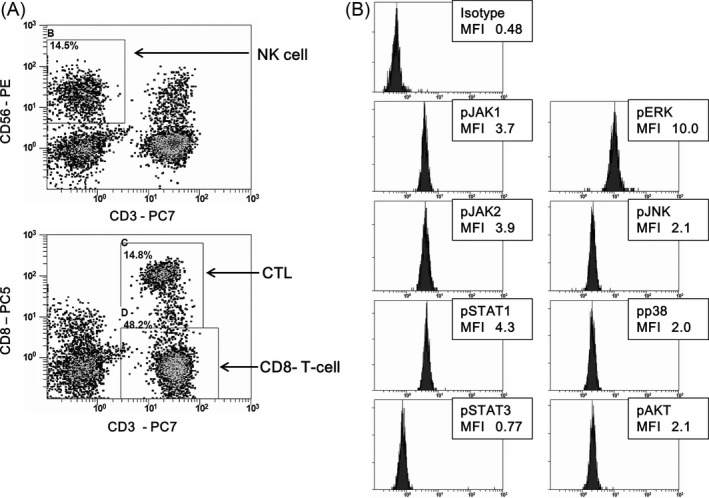
Flow cytometric analysis of each lymphocyte subset and expression of phosphorylated proteins. Representative data are shown. Lymphocyte fractions are classified according to surface antibodies including CD3, CD8, and CD56. Natural killer (NK) cells were defined as the CD3‐CD56^+^ immunophenotype and cytotoxic T lymphocytes (CTLs) were CD3^+^ CD8^+^ (A). Cells were costained with phospho‐specific antibodies, including antibodies targeting pJAK1, pJAK2, pSTAT1, pSTAT3, pERK, pJNK, pp38, and pAKT, and expression levels of the isotype control and phosphorylated proteins in NK cells are presented (B). The values for the isotype control and each phosphorylated protein are shown as the median fluorescence intensity (MFI).

In total, 30 patients with CML in CP were enrolled in this study. Patient characteristics are presented in Table 1. Of 30 patients, 18 were treated with dasatinib (11 first‐line, seven second‐line or later), nine with first‐line imatinib, and three with first‐line nilotinib. CMV IgG was evaluated only in the dasatinib‐treated patients, with a positive result in 13 patients. Regarding the treatment response, 11 patients were in MMR and 17 in DMR. Data after TKI dosage were ineligible in two patients (no. 16 and 30). The representative data of histograms using the phospho‐flow method are shown in Figure 1. Lymphocyte fractions were classified according to surface antibodies including CD3, CD8, and CD56 (Fig. 1A). Cells were costained with phospho‐specific antibodies and the histograms of phosphorylated proteins in NK cell fraction were denoted and expressed as MFI in Figure 1B.

**Table 1:** Patient characteristics enrolled in this study (*n* = 30)

Patientno.	Sex	Age	Treatment	Dose (mg/day)	Response	PE	CMV IgG
1	F	79	Dasatinib, first‐line	50	DMR	Yes	Positive
2	F	74	Dasatinib, first‐line	100	MMR	No	Positive
3	F	34	Dasatinib, first‐line	100	No MMR	No	Negative
4	F	62	Dasatinib, second‐line	100	DMR	Yes	Positive
5	F	31	Dasatinib, second‐line	100	MMR	No	Positive
6	F	66	Dasatinib, second‐line	100	DMR	No	Positive
7	M	27	Dasatinib, first‐line	100	DMR	No	Negative
8	M	25	Dasatinib, first‐line	100	MMR	No	Positive
9	M	64	Dasatinib, first‐line	100	DMR	Yes	Positive
10	M	56	Dasatinib, first‐line	50	DMR	Yes	Positive
11	M	33	Dasatinib, first‐line	100	DMR	No	Positive
12	M	71	Dasatinib, first‐line	50	DMR	Yes	Positive
13	M	25	Dasatinib, first‐line	100	MMR	No	Negative
14	M	30	Dasatinib, first‐line	100	MMR	No	Negative
15	M	34	Dasatinib, second‐line	100	MMR	No	Positive
16	M	47	Dasatinib, third‐line	50	No MMR	No	Positive
17	M	85	Dasatinib, second‐line	50	MMR	Yes	Positive
18	M	30	Dasatinib, second‐line	100	MMR	No	Negative
19	F	38	Imatinib, first‐line	400	DMR	No	NA
20	F	71	Imatinib, first‐line	400	DMR	No	NA
21	F	43	Imatinib, first‐line	400	DMR	No	NA
22	F	63	Imatinib, first‐line	400	DMR	No	NA
23	F	51	Imatinib, first‐line	200	DMR	No	NA
24	F	58	Imatinib, first‐line	400	DMR	No	NA
25	M	68	Imatinib, first‐line	300	MMR	No	NA
26	M	54	Imatinib, first‐line	400	DMR	No	NA
27	M	45	Imatinib, first‐line	400	MMR	No	NA
28	M	72	Nilotinib, first‐line	600	MMR	No	NA
29	M	49	Nilotinib, first‐line	600	DMR	No	NA
30	M	84	Nilotinib, first‐line	600	DMR	No	NA

PE, pleural effusion; CMV, cytomegalovirus; MMR, major molecular response; DMR*,* deep molecular response; NA, not available.

### Mobilization of cytotoxic lymphocytes after TKI intake

**Figure 2:**
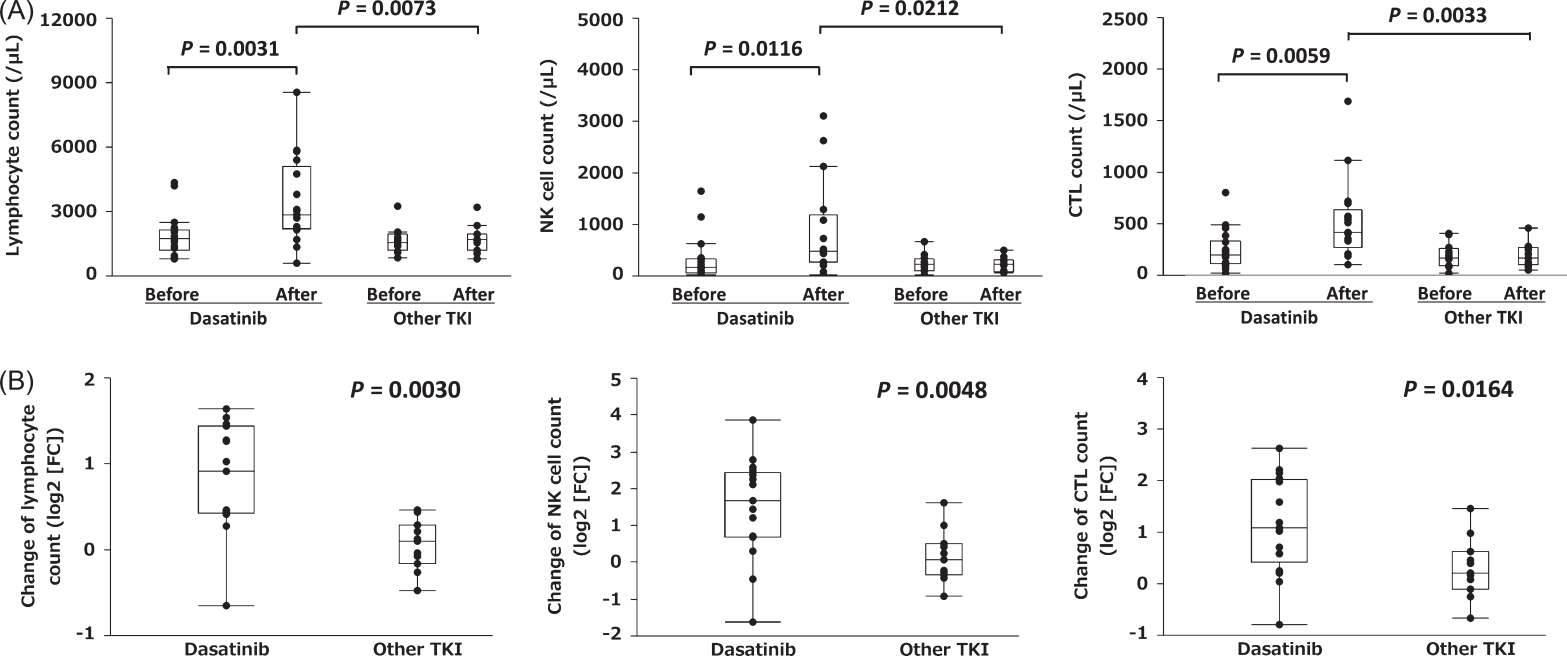
Changes in the number of lymphocytes, natural killer (NK) cells, and cytotoxic T lymphocytes (CTL) are shown. The values were compared before and after treatment (1 h for dasatinib and 2 h for imatinib or nilotinib) or according to treatment (A). The relative changes in the number of lymphocytes, NK cells, and CTLs are also shown and are expressed as log2 (fold change [FC]) (B).

Initially, we determined whether or not cytotoxic lymphocytes were mobilized rapidly after taking dasatinib but not imatinib or nilotinib in the study population. The changes in the absolute values of lymphocytes, NK cells, and CTLs are shown in Figure 2. The number of total lymphocytes, NK cells, or CTLs after dasatinib intake was higher compared to the number before dasatinib intake and the number after taking other TKIs (Fig. 2A). However, the number of total lymphocytes, NK cells, and CTLs before dasatinib intake did not differ statistically from those before intake of other TKIs (Fig. 2A). Furthermore, morphological large granular lymphocytosis was not observed in the blood samples taken before dasatinib intake (data not shown). The relative increase in total lymphocytes, NK cells, or CTLs was apparent only in patients treated with dasatinib, with significant differences compared to those treated with other TKIs (Fig. 2B). Thus, our study population clearly reproduced the rapid lymphocyte mobilization after taking dasatinib, but not imatinib or nilotinib 12.

### Constitutive activities of signal transduction pathways in NK cells and CTLs

**Figure 3:**
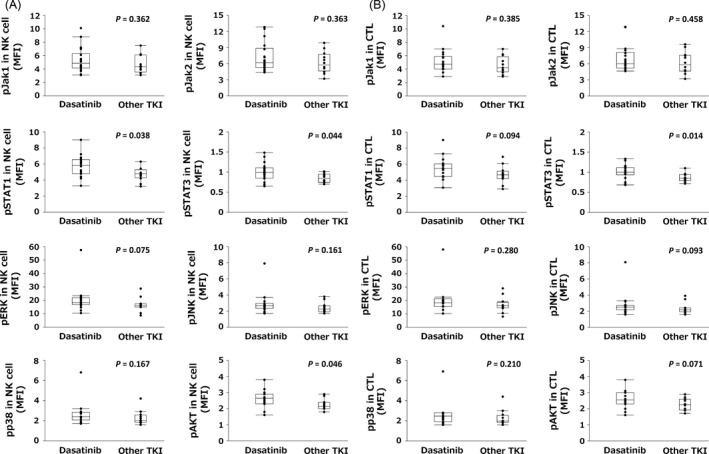
Constitutive levels of phosphorylated proteins including pJAK1, pJAK2, pSTAT1, pSTAT3, pERK, pJNK, pp38, and pAKT in natural killer (NK) cells (A) and cytotoxic T lymphocytes (CTLs) (B) grouped according to treatment (dasatinib [*n* = 18] or other TKI [*n* = 12]) are shown. The values for phosphorylated proteins in each fraction are shown as the median fluorescence intensity (MFI).

We investigated the constitutive levels of phosphorylated proteins in NK cells and CTLs, as shown in Figure 1B. All phosphorylated proteins were detectable and their levels were similar in NK cells and CTLs. With regard to the values obtained, the levels of pSTAT1, pSTAT3, and pAKT in NK cells and pSTAT3 in CTLs before taking TKI were significantly higher in dasatinib‐treated patients than in imatinib‐ or nilotinib‐treated patients (Fig. 3A and B). Thus, the expression levels of some phosphorylated proteins were higher in patients on dasatinib treatment. However, levels of MAPKs, including pERK, pJNK, and pp38, did not differ between groups.

### Change of signal transduction pathways in cytotoxic lymphocytes

**Figure 4:**
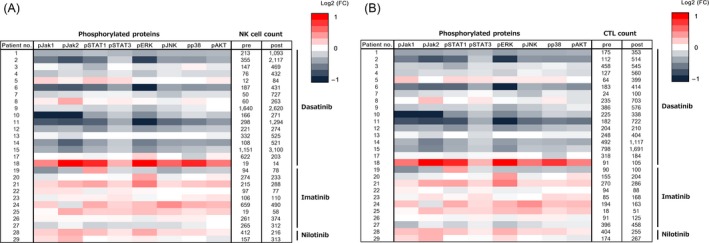
Heat map analysis showing changes in the values in natural killer (NK) cell (A) and cytotoxic T lymphocyte (CTL) (B) fractions (*n* = 28). Changes in the median fluorescence intensity (MFI) values of phosphorylated proteins in each fraction were compared before and after treatment (1 h for dasatinib [patient no. 1–15, 17, 18] and 2 h for imatinib or nilotinib [patient no. 19–29]) and expressed as log2 (fold change [FC]).

Following treatment with TKIs, the change in transduction pathways was analyzed by separating NK cells and CTLs. Results for the 28 available patients (17 dasatinib, nine imatinib, and two nilotinib) are shown in Figure 4. A heat map analysis demonstrated inhibition of signal transduction pathways primarily in dasatinib‐treated patients (no. 1–15, 17, 18) but not in imatinib‐ or nilotinib‐treated (no. 19–29) patients, suggesting that inhibition of signal transduction pathways is the major event occurring in dasatinib‐treated patients. This phenomenon was common to NK cells and CTLs after consumption of dasatinib, but the detailed profiles slightly differed in each patient (Fig. 4A and B). Other factors such as treatment term, age, sex, and treatment line were not associated with a change in cell signaling.

### Correlations between changes in phosphorylated protein levels and mobilization of cytotoxic lymphocytes

**Figure 5:**
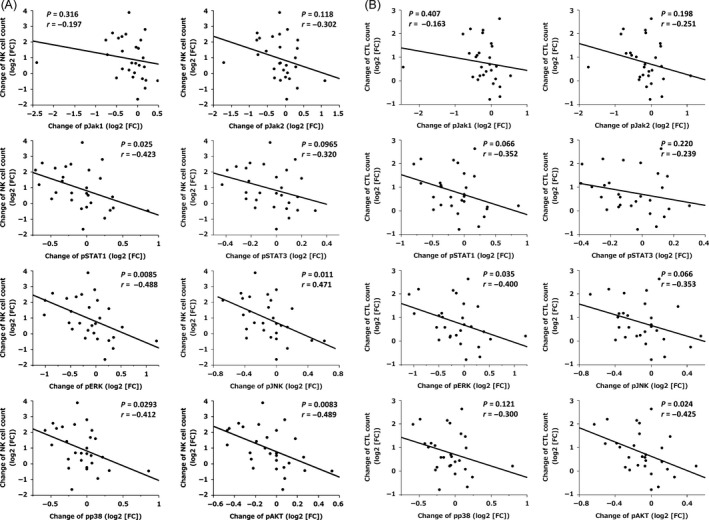
Correlations between changes in cell number and changes in the expression levels of each phosphorylated protein in natural killer (NK) cell and cytotoxic T lymphocyte (CTL) fractions (*n* = 28). Values are shown as log2 (fold change [FC]).

Next, we investigated whether or not inhibition of phosphorylated proteins was correlated with lymphocyte mobilization. As shown in Figure 5, the relative increase in NK cell counts was closely correlated with the magnitude of STAT1, pERK, pJNK, pp38, and pAKT inhibition in NK cell fractions (Fig. 5A). Similarly, the relative increase in CTL counts was also closely correlated with the magnitude of pERK and pAKT inhibition in CTL fractions, suggesting that changes in the expression of the pERK and pAKT pathways are critical for regulating cytotoxic lymphocyte mobilization in both NK cells and CTLs (Fig. 5B).

### Dasatinib directly induces changes in pERK and pAKT

**Figure 6:**
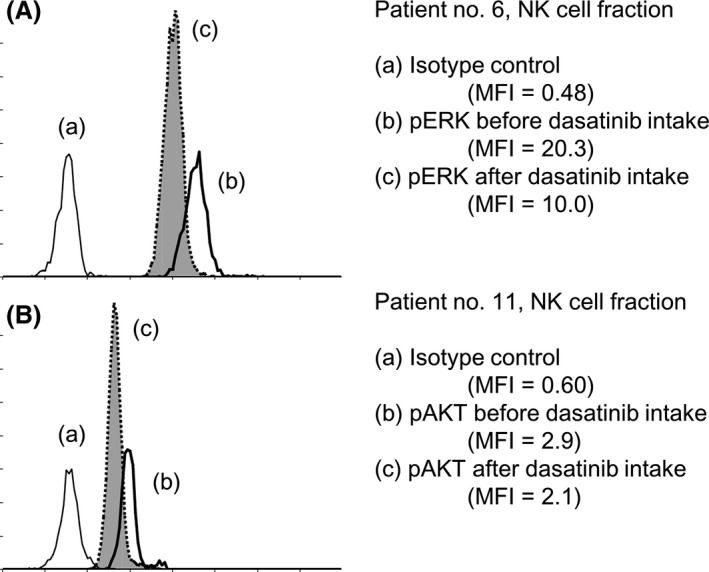
Representative histograms showing expression changes of pERK (A) and pAKT (B) in the natural killer (NK) cell fraction.

**Figure 7:**
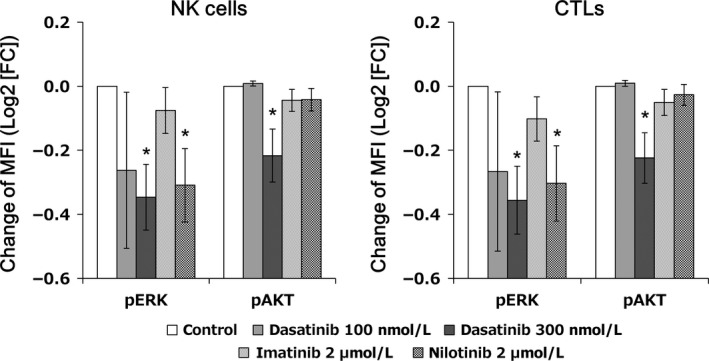
Direct effect of dasatinib on cytotoxic lymphocytes. Mononuclear cells obtained from peripheral blood before taking dasatinib were incubated with dimethylsulfoxide (DMSO) vehicle, 100 nmol/L or 300 nmol/L dasatinib, 2 *μ*mol/L imatinib, or 2 *μ*mol/L nilotinib, for 1 h, fixed with 1.5% paraformaldehyde, and permeabilized with 90% methanol, followed by surface and intracellular staining. The staining was evaluated by flow cytometry. Changes in the expression of phosphorylated proteins, including pERK and pAKT, were evaluated in five samples derived from patients who responded to dasatinib. Levels of each phosphorylated protein were evaluated as the median fluorescence intensity (MFI), and the ratio compared to the control was shown as log2 (fold change [FC]). Results are shown as the mean ± standard error of the mean. *P <* *0.05

It is assumed that lymphocyte mobilization might be involved in the changes in the expression of phosphorylated proteins described above, because differences in phosphorylated protein levels between the mobilized lymphocytes and those already existing in the circulation cannot be measured. In this regard, representative histograms for pERK or pAKT expression before and after dasatinib treatment are shown in Figure 6. A significant inhibition of pERK and pAKT in NK cells after dasatinib intake was observed and the increase in the number of NK cells was a result of lymphocyte mobilization. This finding suggests that the expression of phosphorylated proteins in cytotoxic lymphocytes mobilized by dasatinib from the tissue in which lymphocytes were pooled was almost the same as that in circulating cytotoxic lymphocytes exposed to dasatinib. Moreover, we performed an in vitro study to test whether or not dasatinib directly influences phosphorylated proteins, including pERK and pAKT, in cytotoxic lymphocytes. Mononuclear cells were obtained from patients before taking dasatinib who had lymphocyte mobilization. The cells were incubated with 100 nmol/L or 300 nmol/L dasatinib, 2 *μ*mol/L imatinib, 2 *μ*mol/L nilotinib, or DMSO vehicle for 1 h, fixed with PFA, permeabilized with methanol, stained with each antibody (surface antibodies and antibodies against pERK and pAKT) and evaluated by flow cytometry (*n* = 5). The results are shown in Figure 7. Dasatinib treatment reduced the expression of pERK and pAKT in both NK cells and CTLs with significant differences at a concentration of 300 nmol/L compared to the DMSO control. No significant apoptotic effect was observed in lymphocytes treated with 300 nmol/L dasatinib by trypan blue exclusion assay (data not shown). Thus, pERK and pAKT inhibition results from a direct effect on cytotoxic lymphocytes. However, nilotinib also significantly inhibited pERK expression (but not pAKT) in both NK cells and CTLs.

### Association between the incidence of pleural effusion (PE) and pERK or pAKT inhibition

The major adverse event associated with dasatinib treatment was the incidence of PE, which occurred in six patients (Table 1). With respect to the association of incidence of PE, there were no differences in the levels of pERK and pAKT in NK cells and CTLs. However, all but one PE‐positive patient had a reduced dasatinib dose (50 mg) at the time of sample collection, possibly resulting in an underestimation of the response to dasatinib (data not shown).

### Association of CMV IgG status with pERK and pAKT inhibition

**Figure 8:**
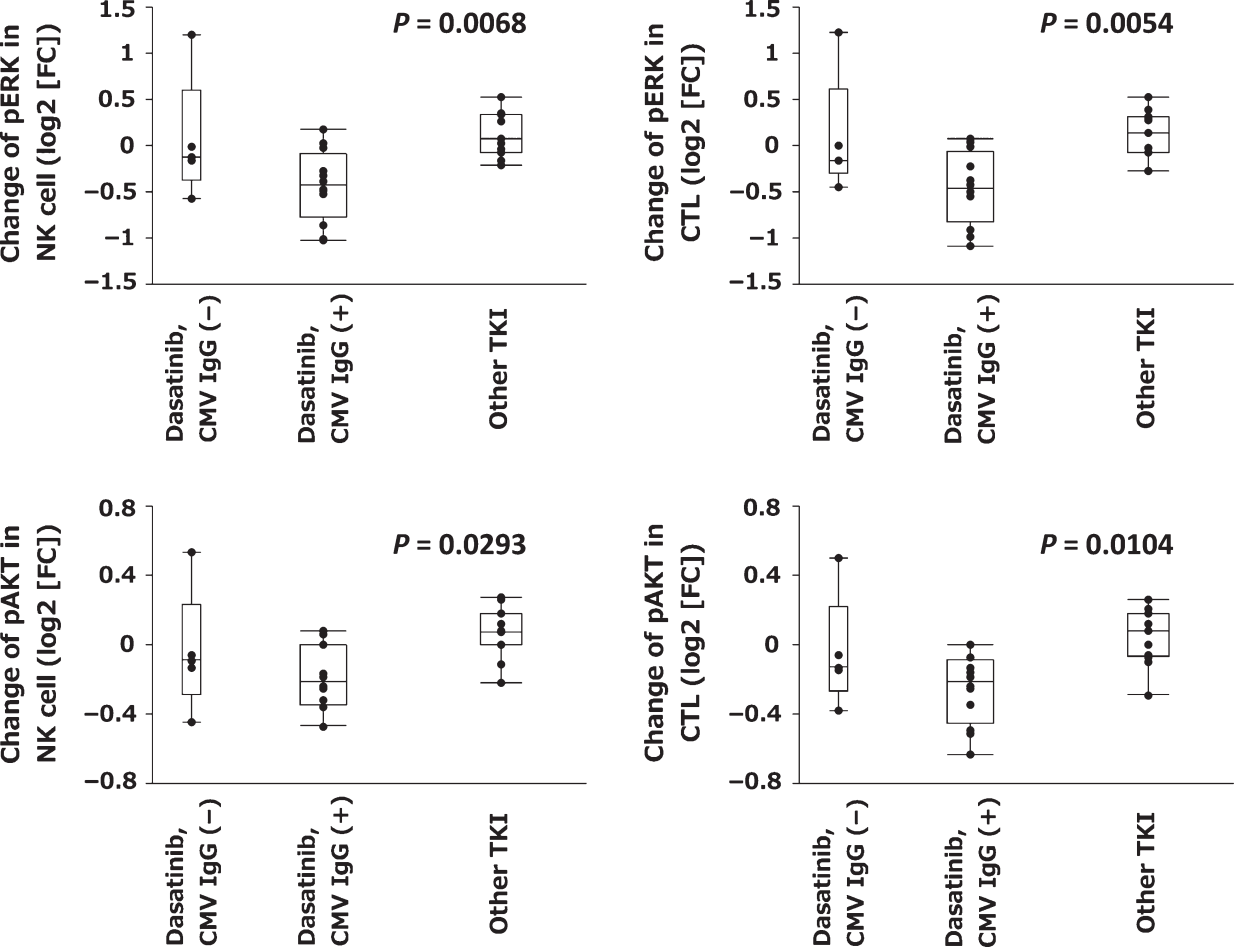
Influence of prior history of cytomegalovirus (CMV) infection on the change of signal transduction pathways. CMV IgG status was examined in patients receiving dasatinib treatment, with positivity in 13 and negativity in five. Eleven patients were treated with other tyrosine kinase inhibitors (TKIs). Changes of pERK and pAKT expression in natural killer (NK) cells and cytotoxic T lymphocytes (CTLs) according to CMV IgG status are shown as log2 (fold change [FC]).

Finally, we analyzed the association between CMV IgG status and pERK or pAKT inhibition after dasatinib intake to clarify whether or not the factors identified were linked to prior CMV infection status. As shown in Figure 8, the effects of pERK or pAKT inhibition on both NK cell and CTL counts were relatively low in CMV IgG‐negative, dasatinib‐treated patients and were similar to those in patients treated with other TKI. Thus, the sensitivity to dasatinib in cytotoxic lymphocytes is more profound in CMV IgG‐positive patients.

## Discussion

A change in signal transduction pathways by TKIs is a major event that influences the incidence of adverse events as well as treatment efficacy in patients. Lymphocytosis is observed in 27–73% of patients under dasatinib treatment and is associated not only with the development of PE, colitis, and autoimmune‐like symptoms but also with favorable outcomes 7, 8, 9, 20. Evidence for the importance of lymphocytosis has accumulated, but the cell signaling events involved in specific phenomena have not yet been elucidated. In this study, we identified for the first time, the cell signaling events for cytotoxic lymphocytes in CML patients treated with dasatinib.

Our study demonstrated that pERK and pAKT expression was significantly reduced in dasatinib‐treated patients compared to imatinib‐ and nilotinib‐treated patients and dasatinib's inhibitory effect on them was closely associated with the mobilization of cytotoxic lymphocytes, but this effect seemed to be linked to a prior history of CMV infection. Further study is required to determine the target molecule affected by dasatinib in patients with CMV IgG positivity. In addition, our in vitro study showed that inhibitory effects on phosphorylated proteins, including pERK and pAKT, were more apparent upon treatment with 300 nmol/L dasatinib than with 100 nmol/L, supporting the fact that the incidence of lymphocytosis depends on drug concentration. Because lymphocytosis actually occurs at concentrations less than 300 nmol/L 12, we assumed that dasatinib might rapidly distribute into tissues where lymphocytes are pooled at higher concentrations than in circulation. Indeed, it has been shown in rat that dasatinib concentration in tissues (such as the lung and liver) was clearly higher than that in circulation after 1 h of exposure to dasatinib 21.

Although association with a Th1 response is assumed in dasatinib‐treated patients as suggested by the predominant expression of interferon‐*γ* in T‐cells 22, the rapid mobilization in cytotoxic lymphocytes is likely not cytokine‐dependent because of the lack of evidence of cytokine secretion after dasatinib intake 12. However, higher levels of pSTAT1 and/or pSTAT3 at constitutive levels in dasatinib‐treated patients might partially result from a Th1 response. In contrast to our study results, a previous study by Jalkansen et al. 19 showed that dasatinib inhibits the level of pSTAT3 in nonmalignant cells. Because our study result showed a transient inhibition of pSTAT3 after dasatinib intake in cytotoxic lymphocytes, the discrepancy was considered to be due to the timing of sample collection. Furthermore, there may be some differences among TKIs in relation to treatment response and the change in the signal transduction pathways. Jalkanen et al. 23 analyzed other signal transduction pathways in mononuclear cells derived from the bone marrow in imatinib‐treated patients and reported that patients who achieved optimal treatment response showed lower expression level of pSTAT5b or phospholipase C‐*γ*. On the other hand, patients treated with dasatinib showed higher levels of pERK and pAKT than those in the patients treated with other TKIs, suggesting the rationale that activated signal transduction pathways are associated with the induction of immune‐mediated mechanisms. The association of treatment response to dasatinib and pERK or pAKT expression at a constitutive level should be investigated in larger‐scale studies.

A transient increase in malignant cells in the circulation in patients with chronic lymphocytic leukemia (CLL) was observed after treatment with Bruton's tyrosine kinase (BTK) inhibitor ibrutinib or phosphoinositide 3‐kinase (PI3K) inhibitor idelalisib 24, 25, similar to the lymphocytosis in dasatinib‐treated patients. BTK and PI3K are essential pathways associated with the mechanism underlying the development and progression of CLL 26, 27. Pharmacologic inhibition of these pathways inhibits downstream pathways, including pERK or pAKT, which are major regulators for integrin expression 15, 16. The underlying mechanism for CLL cell expansion is believed to be the loss of potency to adhere to the microenvironment through cell signaling inhibition. This phenomenon observed in CLL patients supports our finding about inhibitory effects for integrin via signal transduction pathways linked to the mobilization of cytotoxic lymphocytes. Although there is little margin for integrin degradation when the half‐life of integrin molecules is taken into account (approximately 18 h) 28, a direct effect of dasatinib on signal transduction pathways could explain the mechanism underlying the rapid mobilization of lymphocytes, which occurs within 1 h. Integrin heterodimers are constantly internalized from the cell membrane to endosomal compartments in the processes of cell adhesion and migration 29. This traffic system, the so‐called integrin recycling system, has a shorter turnaround time (around 30 min) 30, and integrin recycling from the endosome to the cell surface membrane is positively regulated by ERK and AKT activity 15, 16, 29, 31. In particular, integrin *α*5*β*1, *α*v*β*3, and *α*6*β*4 are regulated by the AKT/GSK‐3*β* pathway 29. Collectively, the expression of integrin on membrane surface could be reduced within 1 h owing to delayed integrin turnover by pERK and/or pAKT inhibition. Therefore, we believe that suppression of the integrin recycling system through the inhibition of pERK and/or pAKT pathways is a key mechanism that enables mobilization of cytotoxic lymphocytes.

However, some evidence has suggested that the proliferated NK cells show a differentiated immunophenotype, and their cytotoxicity is more potent than that of cells not exposed to dasatinib. It is therefore natural to wonder whether inhibition of the signal transduction pathways associated with dasatinib may suppress immune function. Previous reports have shown that lymphocyte expansion is associated with CMV‐associated infection or reactivation 13, but other studies have not 32. This discrepancy may result from differences in the baseline characteristics of the patients investigated. Dasatinib treatment would influence the incidence of infectious complications if used in patients with less active immune function. Furthermore, suppression of immune function in vitro studies is believed to result from maintenance of a consistent dasatinib concentration, as the pharmacokinetics are considerably different from those in vivo 33, 34, 35. In our study, remarkable infectious complications were not observed, probably because of immunocompetence and well‐controlled disease status in the patients analyzed. The constitutive levels in signal transduction pathways investigated in dasatinib‐treated patients were almost the same as or higher than those in patients treated with other TKIs. Taking these results, the treatment schedule (once daily), and a shorter half‐life of dasatinib in the blood into account, pERK or pAKT inhibition is transient, and these kinases are reactivated within a short period of time. Therefore, the expression of these phosphorylated proteins in cytotoxic lymphocytes dramatically and dynamically fluctuates with dasatinib treatment. Based on these findings, we assumed that the effect of dasatinib, similar to pulse therapy, induced an immune response through transient and repeated cell signal inhibition. Interestingly, a previous in vitro study by Hassold et al. 36 demonstrated that NK cells treated with dasatinib showed a reduced effector function but their cytotoxicity was potentiated after washout of dasatinib, supporting our notion that reactivation of those signal transduction pathways along with decreased drug concentration leads to the potentiation of immune function. Collectively, we propose that the response to dasatinib indicated by rapid mobilization of cytotoxic lymphocytes is a result of transient inhibition of signal transduction pathways in the immune system, but the prevalence of the signal response confers the activation of tumor immunity.

In conclusion, our data demonstrating changes in signal transduction pathways in cytotoxic lymphocytes using the phospho‐flow method offer novel insights into the mechanisms underlying dasatinib‐induced cytotoxic lymphocyte mobilization and activation of immune function, which may be highly advantageous because of the potential eradication of minimal residual disease.

## Conflicts of Interest

NI and YH received honoraria and speaker fees from Bristol‐Myers Squibb and Novartis. MT reports research funds from Bristol‐Myers Squibb and Novartis.
